# Impact of AI workplace anxiety on life satisfaction among service industry employees: exploring mediating and moderating factors

**DOI:** 10.3389/fpsyg.2025.1603393

**Published:** 2025-08-04

**Authors:** Zhao Feng, Chun mei Hu, Sheng Chen, Jia yong Xu, Yue Zhang, Miao Hao

**Affiliations:** ^1^School of Literature, Journalism and Communication, Xihua University, Chengdu, China; ^2^Mental Health Education and Counseling Center, Chongqing University of Arts and Sciences, Chongqing, China; ^3^Shenzhen BEEPLUS Technology Co., Ltd., Shenzhen, China; ^4^Chongqing Digital Economy Talent Market, Yongchuan Market, Chongqing, China

**Keywords:** artificial intelligence job anxiety, life satisfaction, negative emotions, social support, service industry employees

## Abstract

**Objective:**

To investigate the impact of artificial intelligence (AI) job anxiety on service industry employees’ life satisfaction and offer insights to mitigate its negative effects.

**Design:**

Cross-sectional study.

**Methods:**

A questionnaire survey was conducted among 600 service employees via the Questionnaire Star platform, with 549 valid responses. PROCESS Models 4 and 7 were used to test mediation and moderated mediation effects.

**Results:**

Life satisfaction was above average. AI job anxiety significantly and negatively predicted life satisfaction (*t* = −3.905, *p* < 0.001), fully mediated by negative emotions (*β* = −0.161, 95% CI = −0.219 ~ −0.107). Social support moderated the effect of AI anxiety on negative emotions (*β* = −0.098, *t* = −3.455, *p* < 0.01).

**Conclusion:**

AI job anxiety reduces employees’ life satisfaction. This effect can be alleviated by enhancing vocational training, emotional regulation, and social support systems.

## Introduction

The wave of artificial intelligence technology has swept across the globe. Currently, significant breakthroughs in deep learning are propelling the transition from the AI 1.0 era to the AI 2.0 era. According to the “Artificial Intelligence Index Report 2023 (AI Index), “compiled by a multidisciplinary group formed by Stanford University’s Human-Centered AI Institute (HAI) in collaboration with academia and industry, China has published the highest number of AI-related papers globally. Although these papers may not match the quality of those from the United States, they nonetheless reflect the immense enthusiasm of the Chinese academic community for the field of artificial intelligence (ScienceNet, 2023). At the 2024 World Artificial Intelligence Conference’s Science Frontier Forum, the “2023 Global Artificial Intelligence Innovation Index Report,” jointly developed by the China Institute of Scientific and Technical Information (CISTI) and Peking University, revealed that a framework of indicators was constructed based on five dimensions: foundational support, resources and environment, scientific and technological research and development, industry and application, and international cooperation and exchange. This report quantitatively assesses the innovation development and governance of artificial intelligence in 46 key countries. The results indicate that, overall, in 2023, the United States and China remain in the top tier of the global artificial intelligence field, with the U.S. scoring 74.71 and China scoring 52.59.

The report points out that, although China has not yet established a core competitive advantage with absolute leadership in the field of artificial intelligence, it has achieved positive results in talent cultivation and industrial development (The Paper, 2023).

Therefore, the development of artificial intelligence in China has also driven reforms across various industries, with the service sector being one of the most affected. Historically, the modern and contemporary development of Chinese society has been referred to as the “Industrious Revolution,” where the population size and social structure provided a relatively inexpensive labor force for labor-intensive industries, which was crucial for the growth of China’s service sector. However, today, the path of the “Industrious Revolution” faces crises such as an aging population and rising labor costs, and artificial intelligence may emerge as a new solution to these challenges (Li, 2017).

Accompanied by the social anxieties triggered by artificial intelligence technology, this has become a focal point of research. Among these, AI workplace anxiety is a noteworthy topic. AI workplace anxiety refers to an individual’s unease and fear regarding the loss of control over artificial intelligence in the workplace, which can provoke negative emotions in employees and subsequently affect their life satisfaction. Life satisfaction is the subjective evaluation of an individual’s overall quality of life, which not only influences personal physical and mental health but also impacts the quality of work (Lin et al., 2023). Numerous studies indicate that various factors such as socioeconomic status, social factors (including social networks and government policies), macroeconomic factors (such as unemployment rates and social welfare), personality traits (such as values and self-esteem), and life events can all impact life satisfaction (Yao, 2020). In China, research on the impact of AI workplace anxiety on life satisfaction is still relatively scarce. However, existing studies predict that in the next 20 years, 76.76% of jobs in China will be affected by artificial intelligence, leading to widespread unemployment anxiety among employees and demonstrating the presence of AI-related anxiety (Chen and Xu, 2018). This issue is poised to have a significant impact on the mental health of Chinese society, social harmony, and the potential for harmonious coexistence between humans and artificial intelligence. As such, it warrants critical attention. Despite growing attention on AI’s workplace impact, limited research has explored how AI-related anxiety affects subjective well-being via emotional and social mechanisms, particularly within China’s service sector. Accordingly, this study investigates whether artificial intelligence has begun to generate job-related anxiety in this sector and how it influences employees’ life satisfaction.

### Technological advancement or technological alienation: research on AI anxiety

The human fear of uncertainty, preference for order and structure, and desire for predictability are instinctive tendencies (Webster and Kruglanski, 1994). Therefore, whenever significant breakthroughs occur in science and technology, voices of anxiety and fear often rise to a fever pitch. On one hand, advancements in AI technology instantly enhance human efficiency, while the remarkable self-learning capabilities of machines in the AI 2.0 era generate considerable excitement. For instance, AI systems like ChatGPT rapidly amassed 123 million monthly active users within just 3 months of their launch (Nature, 2023). On the other hand, as artificial intelligence technology continues to penetrate deeper into human life, issues such as personal data leaks, low-quality information output, fraudulent misuse, and information overload have begun to trigger information anxiety (You, 2023; Zhu et al., 2023; Wang and Zhang, 2023). The development of technology is meant to serve humanity, but technological alienation stands in opposition to humans. Technological alienation is one of the important fulcrums for analyzing the modernity crisis in human society within the field of social economy according to Marx’s theory of alienation. Meanwhile, the employment anxiety of workers is considered one of the key points in discussing technological alienation in the era of artificial intelligence (Xu, 2022). The anxiety stemming from technological alienation is essentially rooted in the personal existential crisis of workers. Although artificial intelligence has not yet widely replaced human jobs, research suggests that the formation and expansion of the private ownership of digital means of production continue to perpetuate and entrench the vulnerable position of workers in the job market under private ownership conditions, becoming the premise and basis for the realization of workers’ employment anxiety in the context of AIGC technology alienation.” (Wang and Su, 2024).

However, in previous studies, researchers have generally exhibited a “Pro-Change Bias” regarding the diffusion of technological innovations, assuming that people are mostly willing to accept change and try innovative products. This assumption serves as a foundation for exploring the extent and reasons for the adoption of innovations (Talke and Heidenreich, 2014). Therefore, classic theories, including the Diffusion of Innovations, Technology Acceptance Model (TAM), and Unified Theory of Acceptance and Use of Technology (UTAUT), adopt a pro-change “positive bias.” These theories focus on the psychological, attitudinal, and behavioral aspects of users’ acceptance of innovative technologies and products, while to some extent overlooking users’ instinctive aversion to the uncertainty, disorder, and ambiguity brought about by innovation.

In reality, for most people, there is a greater tendency to maintain the status quo and consistency rather than pursuing change and innovation. Some studies summarize this inclination as users’ aversion to change and their satisfaction with the current situation (Talke and Heidenreich, 2014). Some researchers have identified six reasons for people’s instinctive rejection of technological innovation: fear of losing control, dogmatism, cognitive rigidity, limited ability to cope with change, intolerance during the adaptation period, weak curiosity, and habitual use (Xiang, 2024).

For contemporary Chinese society, artificial intelligence has not yet fully matured; therefore, not all workers have interacted with or utilized AI, nor do they possess accurate understandings of it. However, due to the “ignorance” stemming from information asymmetry and the “fear” amplified by media portrayals, the pseudo-issues that have not yet been encountered by users during the diffusion of technological innovation may be more complex than the real issues arising from current technologies. This can lead to longer resistance periods in the diffusion of technological innovation (Xiang, 2024).

Therefore, we propose Research Hypothesis 1: Current artificial intelligence technology has already generated workplace anxiety in the service industry.

### Welfare or burden: research on life satisfaction amidst the transformations brought by artificial intelligence technology

Since the emergence of social media, information technology has begun to have a profound impact on people’s social relationships, subsequently affecting their life satisfaction (Pang, 2022). Some studies suggest that this impact may be negative (Kross et al., 2013). Especially for developing countries, the negative impact of these relationship-oriented media may even be manifested in the slowdown of continuous population growth (Hossain and Munam, 2022). The anthropomorphic features of artificial intelligence lead people to easily perceive it as a peer or even a friend during use, fostering interactions based on para-social relationships (Joerling et al., 2019); For example, research results regarding ChatGPT indicate that, in addition to efficiently providing relevant content services, the relevant responses offered by ChatGPT can also meet users’ emotional needs, which are positively correlated with happiness (Aburumman et al., 2024), thereby enhancing their life satisfaction. Research data from the United States has indicated that artificial intelligence is likely to reduce the demand for skilled labor and their wage levels in the short term, further exacerbating wage disparities. This may lead to job polarization, resulting in a decrease in the number of medium-income, medium-skill demand positions (Frey and Osborne, 2017). According to survey data from Frey, there are 702 occupations in the future United States that could potentially be replaced by computers, and 47% of American workers are at risk of employment disruption due to artificial intelligence (Frey and Osborne, 2017).

In China, it is predicted that over 50% of jobs will be impacted by artificial intelligence in the next 20 years. However, research also suggests that due to differences in educational levels, labor skills, and the regions of the workforce (Liu, 2023), the emergence of artificial intelligence may serve as either a complementary or a substitutive force for different occupations (Chen H. Y. et al., 2018; Wei et al., 2021). If some workers perceive AI as a substitutive force that displaces their roles, this could negatively affect their evaluations of job and life satisfaction, as well as reduce their willingness to participate in the labor market (Acemoglu and Restrepo, 2016).

In the service industry, artificial intelligence provides more meaningful interactions in human-machine communication than other forms of robots (Kim et al., 2019). Artificial intelligence can predict user preferences through intelligent algorithms, significantly enhancing the efficiency and accuracy of services (Chung et al., 2016). Artificial intelligence’s cognition, behavior, and emotional expression can more effectively meet the needs of consumers with higher demands for personalization, thereby enhancing users’ well-being and satisfaction (Henkens et al., 2020). For users with high levels of social anxiety, interacting with human service providers may lead to persistent and significant feelings of discomfort, tension, and even fear due to concerns about being scrutinized, among other negative manifestations (Li et al., 2017). In contrast, artificial intelligence service providers can mitigate these negative manifestations. When interacting with robots, users experience a greater sense of self-control, which empowers consumers with high social anxiety to engage in goal-directed motivations and regulate their behaviors (service robots) (Liu et al., 2024).

Although robotic service providers cannot guarantee the emergence of positive psychological outcomes for users, concerns about potential privacy risks associated with robot services, for example, can lead consumers to develop negative usage attitudes (Hsu and Lin, 2016). However, the emergence of artificial intelligence is still seen as a significant challenge to human service industry workers, the more low-skill jobs that do not require special skills or complex thinking, the more susceptible they are to the panic of “machine replacement.”(Brynjolfsson et al., 2024). The social perception, self-evaluation, and coping strategies of workers in these industries may be negatively impacted. For instance, employees facing unemployment anxiety are more likely to focus on the negative aspects of society, prioritize negative information, and overlook positive information. They may lose confidence and maintain low self-esteem, leading them to adopt negative coping strategies such as avoidance and resistance when confronting the challenges posed by artificial intelligence in their work and life, which can affect their mental health. Therefore, we propose Research Hypothesis 2 and 3. Research Hypothesis 2: AI workplace anxiety negatively predicts the life satisfaction of service industry workers. Research Hypothesis 3: Negative emotions mediate the relationship between AI workplace anxiety and life satisfaction.

### Stress reduction and efficiency enhancement: research on social support

The definition of social support can be viewed from both subjective and objective perspectives. From the subjective perspective, social support is considered a form of information that allows individuals to feel that they are cared for, loved, and respected (Cobb, 1976). From the objective perspective, social support is defined as an objective existence or the social interaction relationships that individuals perceive (Sarason et al., 1983). Social support can encompass material support as well as emotional support, which includes psychological comfort. It can also be subdivided into normative support provided by laws and institutions, known as formal support, and non-normative support obtained through social relationships such as marriage, kinship, and community ties (Mu, 2002; Li, 2012). It can be also categorized into three research perspectives: perceived social support, actual social support, and online social support. Actual social support refers to objective and tangible support such as financial aid, instrumental help, or formal institutional support; Online social support refers to interpersonal support exchanged through social media platforms or online communities. Perceived social support is defined as an individual’s perception of the level of social support received. In situations where individuals experience difficulties or stress, the perception of social support can mitigate the negative impact of adverse stimuli on their physical and mental well-being, enhancing positive emotional experiences (Zeng and Lü, 2024; Zeng et al., 2023). Research has also focused on the impact of social support on individuals’ work status. Studies involving various professions, such as nurses, telecommunications employees, and open-pit coal miners, have shown that higher levels of social support are associated with lower negative emotions in the workplace (Xu et al., 2024; Li et al., 2023), This is because social support can positively predict individuals’ self-efficacy, enhancing their positive self-perception in the workplace and leading employees to believe that the organization values their contributions (Ortan et al., 2021; Holzgang et al., 2023). This, in turn, reduces anxiety and depressive emotions experienced at work. It is worth noting that some studies suggest that, compared to Western societies that emphasize individualism, in Chinese society, which advocates collectivism, people tend to place a greater emphasis on group harmony and have a higher need for mutual assistance and reliance, which means that social support has a more significant impact on individuals in collectivist cultures (Xiong et al., 2021). Therefore, we propose Hypothesis 4: Social support moderates the relationship between AI workplace anxiety and negative emotions among workers in the service industry in China.

## Method

### Participants

An online survey was conducted using the professional research platform WJX (Questionnaire Star). The survey targeted service industry employees aged 18 to 58 in Chongqing and Shenzhen, the selection of Chongqing and Shenzhen for this study was strategic for several reasons. Shenzhen, recognized as a Special Economic Zone and a hub for innovation, boasts a rapidly expanding service industry that mirrors the trends of modern economics of China. On the other hand, Chongqing, a pivotal economic and transportation nexus in Western China, presents a varied service sector. Together, these cities represent a diverse range of service industry workers, enhancing the study’s conclusions’ applicability across different contexts. The survey included positions such as supermarket staff, delivery workers, marketing personnel, and restaurant workers. A total of 600 questionnaires were distributed, with 549 valid responses collected, resulting in a response rate of 91.5%. Among the participants, there were 230 males (41.9%) and 319 females (58.1%); 273 individuals (49.7%) were from urban areas, while 276 (50.3%) were from rural areas; 255 participants were married (46.4%), 275 were single (50.1%), and 19 were divorced (3.5%); 312 had no children (56.8%) and 237 had children (43.2%); 291 participants had an education level of college or below (53.0%), while 258 had a bachelor’s degree or higher (47.0%); 292 individuals had a monthly income of less than 5,000 CNY (Chinese Yuan)(53.2%), 193 had an income between 5,000 and 10,000 CNY (25.3%), and 118 had an income between 10,001 and 20,000 CNY (21.5%). The average age was 29.35 ± 8.49 years. All participants signed an informed consent form before participating in the survey. This study was approved by the Ethics Committee of Chongqing University of Arts and Sciences (Approval No. CQWLDF0026).

### Procedure

The China Service Trade Association contacted service industry enterprises in Chongqing and Shenzhen to conduct the survey among their employees; Company management provided employees with the link to the online questionnaire and requested them to complete it. This study employs a cross-sectional survey design, aiming to examine the predictive relationship between AI job anxiety and life satisfaction, as well as the mediating and moderating effects involved A questionnaire survey is used, which consists of three parts. The first part is the “Informed Consent Form, “which informs participants about the purpose, content, anonymity, and confidentiality of the survey. Participants are asked to voluntarily sign the informed consent form, and upon signing, they will proceed to the formal questionnaire. The second part covers demographic variables, including gender, household registration, education level, whether the participants have children, marital status, and monthly income. The third part includes the survey instruments: Satisfaction with Life Scale, Artificial Intelligence Anxiety Scale (AIAS), the Depression Anxiety Stress Scales (DASS) and the Multidimensional Scale of Perceived Social Support (MSPSS).

### Measures

#### Satisfaction with life scale (SWLS)

The scale, developed by Diener, consists of 5 items that measure individuals’ overall satisfaction with their life (Pavot and Diener, 1993). Responses are rated on a 5-point Likert scale, ranging from ‘1 - Strongly Disagree’ to ‘5 - Strongly Agree,’ with higher total scores indicating greater life satisfaction. In this study, the Cronbach’s *α* coefficient for this scale was 0.884.

#### Artificial intelligence anxiety scale (AIAS)

The study employed the Artificial Intelligence Anxiety Scale developed by Li and Huang (2020), which includes 6 items measuring “job replacement anxiety” and “work-related learning anxiety.” These items assess individuals’ anxiety regarding the potential for artificial intelligence to replace their jobs and the difficulties associated with work-related learning. Responses are rated on a 5-point Likert scale, ranging from “1 - Strongly Disagree” to “5 - Strongly Agree” with higher total scores indicating greater levels of AI workplace anxiety. In this study, the Cronbach’s *α* coefficient for this scale was 0.824.

#### Depression anxiety stress scale (DASS)

The Depression Anxiety Stress Scales, originally developed by Lovibond and colleagues, and later refined by Xi Xu (Gong et al., 2010), comprises 21 items. It utilizes a 4-point Likert scale for responses, from “1–Strongly Disagree” to “4–Strongly Agree,” with higher scores reflecting higher levels of negative emotion. In the context of this study, the scale demonstrated a Cronbach’s α coefficient of 0.963.

#### Perceived social support scale (PSSS)

The scale, developed by Zimet et al. (1988) consists of 12 items and includes three subscales: Family Support, Friend Support, and Other Support. It measures the extent of support individuals perceive from family, friends, and other sources (mainly colleagues, supervisors, and relatives). Responses are rated on a 5-point Likert scale, ranging from ‘1–Strongly Disagree’ to ‘5–Strongly Agree,’ with higher total scores indicating greater levels of perceived social support. In this study, the Cronbach’s α coefficient for this scale was 0.952.

### Statistical analysis

Data entry and analysis were performed using SPSS 23.0. Independent samples *t*-tests and analysis of variance (ANOVA) were conducted to examine demographic differences in life satisfaction among service industry employees. Regression analysis was employed to investigate the predictive relationship between AI workplace anxiety and life satisfaction. The mediation effect of negative emotions and the moderating effect of social support were assessed using Model 4 and Model 7 from the Process macro. To evaluate potential common method bias among the variables, Harman’s single-factor test was applied. The results revealed that the first common factor accounted for 26.95% of the variance, which is below the critical threshold of 40%, indicating that the data in this study are not affected by bias due to similar research methods. A significance level of *p* < 0.05 was set to determine statistical significance.

## Result

### Basic situation of AI workplace anxiety

The total score of AI workplace anxiety among service industry employees was (2.75 ± 0.72). There were no statistically significant differences in AI workplace anxiety across employees with different demographic characteristics (*p* > 0.05). See Table 1.

**Table 1:** The differences of life satisfaction of artificial intelligence job anxiety with different demographic variables.

Demographic variable	Group	AI workplace anxiety	t/*F* value	*p*-value
Gender	Male	2.74 ± 0.73	−0.375	0.708
	Female	2.75 ± 0.71		
Household Registration	Urban	2.72 ± 0.67	−1.085	0.278
	Rural	2.78 ± 0.75		
Education level	College Diploma and Below	2.81 ± 0.74	0.05	0.962
	Bachelor’s Degree and Above	2.81 ± 0.73		
Having children	No	2.74 ± 0.72	−0.097	0.922
	Yes	2.75 ± 0.71		
Marital status	Married	2.72 ± 0.72	0.720	0.487
	Single	2.76 ± 0.71		
	Divorced	2.91 ± 0.64		
Monthly income	<5,000 CNY	2.76 ± 0.71	1.143	0.319
	5,000–10,000 CNY	2.79 ± 0.76		
	>10,000 CNY	2.66 ± 0.59		

### Basic situation of life satisfaction

The overall life satisfaction score for service industry employees is (3.10 ± 0.85). There are statistically significant differences in life satisfaction based on educational level, marital status, having children, and monthly income (*p* < 0.01). Employees with a bachelor’s degree or higher have higher life satisfaction than those with a college diploma or lower; married individuals have higher satisfaction than unmarried or divorced individuals; those with children report higher satisfaction than those without; and employees with a monthly income of 10,000 CNY or more have higher satisfaction than those earning below 10,000 CNY. There are no statistically significant differences in life satisfaction between different genders or household registration status (*p* > 0.05). See Table 2.

**Table 2:** The differences of life satisfaction of service industry employees with different demographic variables.

Demographic variable	Group	Life satisfaction	t/F value	*p*-value
Gender	Male	3.05 ± 0.89	−0.984	0.325
	Female	3.13 ± 0.82		
Household registration	Urban	3.12 ± 0.85	0.674	0.501
	Rural	3.07 ± 0.86		
Education level	College Diploma and Below	2.99 ± 0.89	−2.901***	0.004
	Bachelor’s Degree and Above	3.20 ± 0.81		
Having children	No	2.99 ± 0.80	−3.443**	0.000
	Yes	3.23 ± 0.89		
Marital status	Married①	3.24 ± 0.87	6.664***, ① > ②, ① > ③	0.001
	Single②	2.97 ± 0.81		
	Divorced③	2.98 ± 0.99		
Monthly income	<5000CNY①	2.93 ± 0.86	13.155***, ② > ①, ③ > ①	0.000
	5,000-10000CNY②	3.30 ± 0.77		
	>10000CNY③	3.28 ± 0.84		

* Indicates *p* < 0.05, ** indicates *p* < 0.01, and *** indicates *p* < 0.001. Same below.

The results of the correlation analysis indicate that life satisfaction among service industry employees is significantly negatively correlated with AI workplace anxiety and negative emotions (*r* = −0.170, −0.413, *p* < 0.01), and significantly positively correlated with social support (*r* = 0.514, *p* < 0.01). AI workplace anxiety is significantly positively correlated with negative emotions (*r* = 0.369, *p* < 0.01) and significantly negatively correlated with social support (*r* = −0.174, *p* < 0.01). See Table 3.

**Table 3:** Correlation analysis of major variables.

Variable	M ± SD	①	②	③	④
AI Workplace anxiety ①	2.75 ± 0.72	1			
Negative emotions ②	1.57 ± 0.61	0.369**	1		
Social support ③	3.52 ± 0.84	−0.174**	−0.469**	1	
Social support ④	3.10 ± 0.85	−0.170**	−0.413**	0.514**	1

### Regression analysis of AI workplace anxiety on life satisfaction

Using the educational level, marital status, having children, monthly income, and AI workplace anxiety—factors that showed significant differences in life satisfaction from the univariate analysis—as independent variables, and life satisfaction as the dependent variable, a multiple linear regression analysis was conducted. It was found that monthly income, having children, and AI workplace anxiety together predicted 7.2% of the variance in life satisfaction among service industry employees. Notably, AI workplace anxiety significantly negatively predicted life satisfaction (*t* = −3.905, *p* < 0.001). See Table 4.

**Table 4:** Regression analysis of artificial intelligence job anxiety on life satisfaction.

Independent variable	*R*	*R^2^*	*ΔR^2^*	*β*	*t*	*P*
Monthly income	0.190	0.036	0.034	0.190	4.523	0.000
AI workplace anxiety	0.249	0.062	0.059	−0.162	−3.905	0.000
Having children	0.269	0.072	0.067	0.103	2.406	0.016

### Test of the mediating effect of negative emotions

AI workplace anxiety, negative emotions, and life satisfaction among service industry employees are significantly correlated with each other, thereby meeting the criteria for mediating effect analysis. All variables were centered, and demographic variables demonstrating significant differences in life satisfaction—namely educational level, marital status, number of children, and monthly income—were incorporated as control variables. Employing AI workplace anxiety as the independent variable, negative emotions as the mediating variable, and life satisfaction as the dependent variable, the mediating effect of negative emotions was examined using Model 4 from the Process macro.

The results indicated that the direct effect of AI workplace anxiety on life satisfaction was not statistically significant (*p* > 0.001). In contrast, the direct effect of AI workplace anxiety on negative emotions was significant (*p* < 0.001), with an effect size of 0.301. The mediating effect of AI workplace anxiety on life satisfaction through negative emotions was estimated at −0.161, with a 95% confidence interval (CI) of (−0.219 to −0.107) that does not encompass zero, thereby suggesting that negative emotions serve as a complete mediator between AI workplace anxiety and life satisfaction. The total effect was −0.188, with the mediating effect accounting for 85.64% of the total effect. See Table 5.

**Table 5:** Testing the mediating effect of negative emotion.

Pathway	Direct effect	Mediating effect	*T*	95%*CI*	*p-value*
AI workplace anxiety → Life satisfaction	−0.027	—	−0.551	−0.124 ~ 0.070	0.582
AI workplace anxiety → Negative emotions	0.301	—	8.942	0.235 ~ 0.367	0.000
Negative emotions → Life satisfaction	−0.536	—	−9.121	−0.651 ~ −0.420	0.000
AI workplace anxiety → Negative emotions → Life satisfaction	—	−0.161	—	−0.219 ~ −0.107	—

### Moderated mediation effect test

Given that negative emotions fully mediate the relationship between AI workplace anxiety and life satisfaction among service industry employees, Model 7 from the Process macro was employed to examine whether social support moderates the relationship between AI workplace anxiety and negative emotions. The demographic variables of educational level, marital status, having children, and monthly income were included as control variables, with AI workplace anxiety as the independent variable, negative emotions as the mediating variable, social support as the moderating variable, and life satisfaction as the dependent variable to test the moderated mediation model.

The results indicated that the interaction term between AI workplace anxiety and social support significantly predicted negative emotions (*β* = −0.098, *t* = −3.455, *p* < 0.01), suggesting that social support moderates the effect of AI workplace anxiety on negative emotions. See Table 6.

**Table 6:** Testing the moderated mediation effect.

Result variable	Predictor variable	*R*	*R* ^2^	*F*(df)	*β*	*t*	LICI	ULCI
Negative emotions		0.581	0.338	39.468 (7)				
	Educational level				−0.071	−1.471	−0.166	0.024
	Marital status				0.094	1.964	0.000	0.189
	Number of children				0.068	1.239	−0.040	0.176
	Monthly income				−0.048	−1.530	−0.109	0.014
	AI workplace anxiety				0.602	5.574***	0.390	0.814
	Social support				−0.039	−0.491	−0.193	0.116
	AI workplace anxiety × Social support				−0.098	−3.455**	−0.154	−0.042
Life satisfaction		0.446	0.199	22.424 (6)				
	Educational level				0.011	0.143	−0.136	0.157
	Marital status				−0.023	−0.318	−0.169	0.122
	Having children				0.155	1.845	−0.010	0.321
	Monthly income				0.113	2.350*	0.018	0.207
	AI workplace anxiety				−0.027	−0.551	−0.124	0.070
	Negative emotions				−0.536	−9.121***	−0.651	−0.420

**Figure 1:**
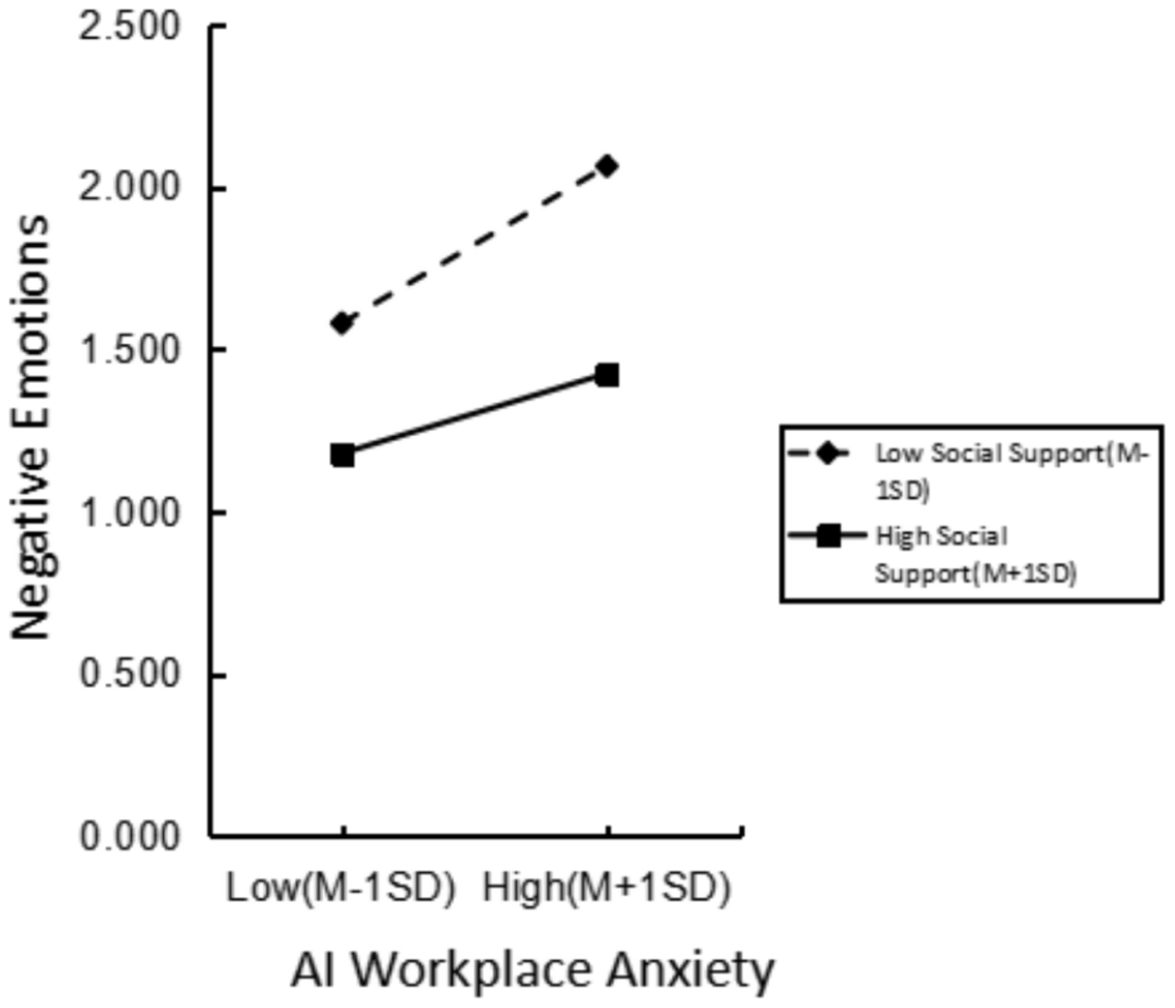
The moderating effect of social support between artificial intelligence job anxiety and negative emotion.

To further analyze the moderating effect of social support on the relationship between AI workplace anxiety and negative emotions among service industry employees, simple slope analysis was conducted by categorizing social support into high and low groups, using one standard deviation above and below the mean. The results indicated that as the level of social support increased, the predictive effect of AI workplace anxiety on negative emotions weakened (from *β* = 0.338, *t* = 8.275, *p* < 0.001 to *β* = 0.173, *t* = 4.714, *p* < 0.001). Higher levels of social support reduced the impact of AI workplace anxiety on negative emotions. See Figure 1.

## Discussion

The results of the survey indicate that the AI workplace anxiety of service industry employees is at a moderate level, with no significant differences across employees with different demographic characteristics. This indicates that service industry workers generally experience anxiety caused by the “impact of AI on jobs,” thus confirming Hypothesis 1: Current artificial intelligence technology has already generated workplace anxiety in the service industry.

Additionally, the survey results revealed that the life satisfaction of service industry employees is at a moderately high level, above the average. Employees with a bachelor’s degree or higher reported greater satisfaction than those with a college diploma and below; married individuals scored higher than unmarried or divorced individuals; employees with children reported greater satisfaction than those without children; and those earning above 10,000 RMB per month showed significantly higher satisfaction than those with monthly incomes below 10,000 RMB. These findings suggest that interventions targeting life satisfaction should be tailored to the specific characteristics of different service industry employee groups. In addition to general interventions, it is essential to strengthen support for employees with lower education levels, those without children, unmarried/divorced individuals, and those with lower incomes.

AI workplace anxiety negatively predicts the life satisfaction of service industry employees. However, after introducing negative emotions as a mediating variable, its direct predictive effect becomes non-significant. This indicates that the negative impact of AI workplace anxiety on life satisfaction primarily stems from the generation of negative emotions such as depression and anxiety. 1. Job substitution effect. The application of AI technology in the service industry can directly replace certain job positions, particularly those with low entry barriers and high levels of repetition, which are at greater risk of being replaced by AI. In populous, developing countries like China, the workforce in these positions is substantial. Robin Li, CEO of Baidu, one of China’s largest internet giants, has suggested that Baidu’s AI technology enables any new hire to immediately possess the communication skills of top salespeople. Additionally, AI service providers can effectively avoid human weaknesses, with “no greed, no fear” and “unlimited energy, able to work 24/7” (Li, 2017), leading employees to face unemployment or an increased risk of unemployment. 2. Deskilling effect. The application of AI technology can result in employees’ professional skills being replaced by automation or downgraded to low-skill tasks that require neither complex thinking nor specialized skills. This can reduce labor costs for certain service roles to as little as one-twentieth of a human worker’s cost, or even zero, thereby weakening employees’ bargaining power and lowering their income levels. 3. Creation effect. The implementation of AI technology can introduce new demands on job positions or generate entirely new roles, increasing the pressure on employees to learn new knowledge and technologies (Wan et al., 2014). This pressure from AI-driven changes can directly lead to negative emotions, such as anxiety, depression, and panic (Wan et al., 2014). Furthermore, when AI workplace anxiety causes negative emotions among service industry employees, these adverse emotional states lead employees to view society and themselves pessimistically. They may focus on negative social information and hold negative self-evaluations, which in turn reduces their life satisfaction (Chen H. Y. et al., 2018). Therefore, Hypotheses 2 and 3 were confirmed: AI workplace anxiety directly and negatively predicts employees’ life satisfaction. At the same time, the impact of AI workplace anxiety on life satisfaction is primarily due to negative emotions.

The survey results show that social support can moderate the effect of AI workplace anxiety on negative emotions among service industry employees, consistent with findings from studies on various groups, including the general public, university students, nurses, telecom employees, and open-pit coal miners. Conservation of Resources Theory suggests that social support is a crucial psychosocial resource for individuals, offering not only material support but also enhancing one’s sense of purpose and goal orientation, promoting positive self-evaluation, and helping to buffer the negative impacts of life stressors and challenges. The stress-buffering model indicates that social support acts as a ‘buffer’ between stress and mental health. Strong social support provides individuals with both material resources and emotional comfort, increasing their effective resources to cope with negative stress events, alleviating the adverse effects of stress, and strengthening social adaptability and psychological well-being (Schwarzer and Knoll, 2007). Thus, when service industry employees face work-related stress brought on by AI technology, strong social support can serve as a crucial psychosocial resource, alleviating the negative emotional impact of workplace anxiety. For individuals with high social support, resources from family, friends, and society help buffer the anxiety caused by AI, reducing the likelihood of negative emotional responses. Conversely, employees with low social support, who lack sufficient psychosocial resources, are more likely to experience negative emotions when dealing with the impact of AI on their work. The collectivist values prevalent in Chinese society intensify the need for group support among individuals (Li et al., 2024). Moreover, regardless of the level of AI workplace anxiety, individuals with higher levels of social support experience lower negative emotions compared to those with lower levels of social support. This indicates that social support can effectively mitigate the increase in negative emotions caused by AI workplace anxiety. Therefore, our proposed Hypothesis 4 is confirmed: strong social support can alleviate the negative emotions resulting from AI workplace anxiety among service industry employees.

Compared with recent studies (e.g., Wang and Su, 2024; Xu, 2022; Holzgang et al., 2023), this research empirically validates the affective mechanism underlying AI workplace anxiety by confirming that negative emotions fully mediate its impact on life satisfaction. Furthermore, it extends the theoretical framework of technological alienation by demonstrating that social support plays a critical moderating role, acting as a buffer against stress induced by AI-related uncertainty. These findings enrich our understanding of the psychological effects of technological change from an emotional and relational perspective.

## Conclusion, strategies and research limitations

In summary, AI workplace anxiety primarily affects service industry employees’ life satisfaction by inducing negative emotions, while social support can alleviate the increase in negative emotions caused by AI workplace anxiety. Based on this, the service industry can take the following measures to mitigate the negative impacts of AI technology on employees and improve their life satisfaction: First, strengthen employee vocational training by increasing educational investments in labor cost and providing job training that aligns with AI technology to enhance employees’ job adaptability (Huang, 2022). Machines replacing humans’ from a ‘displacement’ model to a ‘complementary’ co-employment mindset characterized by harmonious human-machine coexistence. Through effective communication and education, we can mitigate the negative emotions associated with ‘pseudo-issues’ of AI, which are often fueled by information asymmetry and media sensationalism. This approach can help alleviate employees’ anxiety related to artificial intelligence.; Secondly, implement mental health training to enhance employees’ skills in stress management and emotional regulation, enabling them to maintain a healthy work and psychological state. Additionally, organize employee activities and team-building initiatives while simultaneously strengthening managerial support and concern for employees. These efforts aim to improve employees’ perceptions of social support within the organization, thereby enhancing their sense of self-efficacy and positive self-concept. Thirdly, strengthen the development of social security systems (Cao, 2023). To ensure employees’ needs related to unemployment, healthcare, and retirement, companies can also provide more opportunities for interaction with family and the community, such as organizing family day events, thereby enhancing employees’ perceptions of social support from sources outside the organization. This study acknowledges certain limitations: participant responses may be subject to recall bias or reporting bias, and the sample covers a limited number of cities, thus necessitating caution when generalizing the results. Future studies should expand their sample size to further validate the findings.

## Data Availability

The original contributions presented in the study are included in the article/supplementary material, further inquiries can be directed to the corresponding author/s.
