# Correction: Impact of AI workplace anxiety on life satisfaction among service industry employees: exploring mediating and moderating factors

**DOI:** 10.3389/fpsyg.2025.1684157

**Published:** 2025-09-09

**Authors:** Zhao Feng, Chun mei Hu, Sheng Chen, Jia yong Xu, Yue Zhang, Miao Hao

**Affiliations:** ^1^School of Literature, Journalism and Communication, Xihua University, Chengdu, China; ^2^Mental Health Education and Counseling Center, Chongqing University of Arts and Sciences, Chongqing, China; ^3^Shenzhen BEEPLUS Technology Co., Ltd., Shenzhen, China; ^4^Chongqing Digital Economy Talent Market, Yongchuan Market, Chongqing, China

**Keywords:** artificial intelligence job anxiety, life satisfaction, negative emotions, social support, service industry employees

In the published article, the affiliations of authors Zhao Feng and Chun mei Hu were erroneously swapped:

Zhao Feng^1†^

^1^Mental Health Education and Counseling Center, Chongqing University of Arts and Sciences, Chongqing, China

Chun mei Hu^2*†^

^2^School of Literature, Journalism and Communication, Xihua University, Chengdu, China

The correct affiliations are:

Zhao Feng^1†^

^1^School of Literature, Journalism and Communication, Xihua University, Chengdu, China

Chun mei Hu^2*†^

^2^Mental Health Education and Counseling Center, Chongqing University of Arts and Sciences, Chongqing, China

The original version of this article has been updated.

